# Identification of SLC31A1 as a prognostic biomarker and a target for therapeutics in breast cancer

**DOI:** 10.1038/s41598-024-76162-x

**Published:** 2024-10-24

**Authors:** Hongtao Fu, Shanshan Dong, Kun Li

**Affiliations:** 1 Department of Breast Surgery, Hunan Cancer Hospital/the Affiliated Cancer Hospital of Xiangya School of Medicine, Central South University, Changsha, 410006 Hunan China; 2 Department of Breast Surgery, The First Affiliated Hospital with Nanjing Medical University, Nanjing, 210000 China; 3 Department of Medicine, Hunan Cancer Hospital/the Affiliated Cancer Hospital of Xiangya School of Medicine, Central South University, Changsha, 410000 China; 4 Department of Emergency Medicine, The Affiliated Changsha Central Hospital, Hengyang Medical School, University of South China, 161 Shaoshan South Road, Changsha, 410000 China

**Keywords:** Cuproptosis-related genes, Bioinformatics analysis, Biomarker, Breast cancer, Breast cancer, Metastasis

## Abstract

Copper-induced cell death is regulated through protein lipoylation, which is critical for gene expression and phenotypic regulation. Neverless, the role of Cuproptosis-related genes in breast cancer (BC) remains unknown. This study aimed to construct a prognostic signature based on the expression of Cuproptosis-related genes in order to guide the diagnosis and treatment for BC. Cuproptosis-related genes prognostic signature has ata of 1250 BC tissues and 583 normal breast tissues were obtained from The Cancer Genome Atlas (TCGA), Genotype Tissue Expression (GTEx), and GEO GSE65212. The prognostic signature was established and evaluated with nineteen Cuproptosis-related genes. A series of in silico analyses based on SLC31A1, included expression analysis, independent prognostic analysis, correlation analysis, immune-related analysis and survival analysis. Finally, a series of cell experiments (including quantitative real-time polymerase chain reaction and western blot), and mice experiments were applied to evaluate the impact of SLC31A1 on BC. Cuproptosis-related genes prognostic signature has good predictive promising for survival in BC patients. We discovered that SLC31A1SLC31A1 was overexpressed in BC and was its independent prognostic factor. High expression of the SLC31A1 was correlated with poor prognosis and immune infiltrating of BC. SLC31A1 expression is associated with immune, chemotherapeutic and targeted therapy outcomes in BC. The proliferation, migration, and invasiveness of Her2 + enriched BC cells were decreased by SLC31A1 knockdown, also resulting in a decrease in tumor volume in mouse model. SLC31A1 is a candidate biomarker or therapeutic target in precision oncology, with diagnostic and prognostic significance in BC.

**Supplementary Information:**

The online version contains supplementary material available at 10.1038/s41598-024-76162-x.

## New and noteworthy

Copper-induced cell death is mediated mechanism by protein lipoylation, plays an important role in regulating gene expression and phenotype. Our results systematically demonstrated the expression and prognostic value of Cuproptosis-related genes in breast cancer and its key gene **SLC31A1**SLC31A1 to guide diagnosis and treatment for breast cancer. We evidenced through a series of crucial in vivo and in vitro investigations that SLC31A1 has a significant impact on the promotion of Her2 + enriched breast cancer cells.

## Background

Breast cancer (BC) is one of the leading causes of mortality in women throughout the world^1^. Despite great progress in diagnosis, treatment and prognosis for BC patients, especially triple-negative BC patients and advanced BC patients, there still exists poor prognosis due to lack of therapeutic targets as well as limited treatment options^2^. Therefore, more effective biomarkers and disease classification are required for BC. Copper ionophore-induced cell death is a novel cell death pathway that is distinctly different from traditional cell death types, such as apoptosis, ferroptosis, and necrosis^3^.

Copper is essential for living organisms^4^. Copper can be distributed in the cytoplasm through the secretory pathway or with the help of copper chaperones, and it can also be transported to mitochondria with the help of some proteins^5^. This is the main route by which copper is distributed in the cytoplasm^5^. Cancer progression is associated with increased cellular copper concentrations, and cancerous proliferation, angiogenesis, and metastasis all have a defined copper requirement^4^. Copper transporter 1 (CTR1, or hCtr1 encoded by SLC31A1) is a vital copper influx transporter for the transfer of high-affinity copper (possibly reduced Cu I) to cancer cells^6,7^. SLC31A1, as a copper importer, in cells overexpressing SLC31A1, leads to cell death only through the copper-induced pathway^3^.

The latest research uncovers markers of copper-induced cell death^3^. Elesclomol is a copper ionophore that can aid patients whose tumors depend on mitochondria for energy generation^3^, and it has the potential to cure a variety of malignancies that are vulnerable to copper death^3^, including those that higher expression SLC31A1.

We obtained 19 Cuproptosis-related genes from previous literature (FDX1, LIAS, LIPT1, DLD, DLAT, PDHA1, PDHB, MTF1, GLS, CDKN2A, SLC31A1, ATP7A, ATP7B, LIPT2, DBT, DLST, GCSH, NFE2L2, NLRP3)^3,8–10^. The role including clinical relevance and prognostic significance of Cuproptosis-related genes in BC remains unknown^3^. We downloaded RNA-sequencing expression profiles and corresponding clinical information of BC samples from TCGA (https://portal.gdc.com). Based on the method of lasso regression, dimensionality reduction was performed and a prognostic model was constructed.

We included all 19 Cuproptosis-related genes into the analysis of this prognostic model. Combining the results of univariate and multivariate COX analysis, survival analysis and nomogram, we found that SLC31A1 may be the most critical gene among the 19 Cuproptosis-related genes in BC.

We included all 19 Cuproptosis-related genes into the analysis of this prognostic model. After Kaplan–Meier (KM) survival analysis, only SLC31A1, ATP7B, and PDHA1 showed survival differences among Cuproptosis-related genes in BC. However, univariate and multivariate cox regression analyzes of P values showed that only SLC31A1 (*P* < 0.05) could be used as an independent predictor. Furthermore, the 2-, 3-, and 5-year overall survival of BC patients could be predicted using the SLC31A1-based nomogram. Combining the above results of univariate and multivariate COX analysis, survival analysis and nomogram, we found that SLC31A1 may be the most critical gene among 19 Cuproptosis-related genes in BC.

This study aimed to construct a prognostic signature and analysis of key genes (SLC31A1) associated with BC by Cuproptosis-related genes to predict the prognostic outcome of BC patients and guide the diagnosis and treatment of BC. Subsequently, we explored the noncoding RNA (ncRNA)-related regulation of SLC31A1, including microRNAs (miRNAs) and long noncoding RNAs (lncRNAs). We also determined the expression of SLC31A1 to be immunologically correlated in BC. The results showed the relationship between ncRNAs-mediated SLC31A1 and poor prognosis and tumor immune infiltration in BC patients.

## Methods

### Data collection

Obtained RNA sequence expression profile and clinical information for BC using TCGA and GTEx. RNAseq data and corresponding clinical information for 1097 BC were obtained from TCGA dataset (https://portal.gdc.com)^11^. Genotype tissue expression data from 572 healthy tissues were obtained from GTEx (https://gtexportal.org/home/datasets)^12,13^. We supplemented the expression data of 153 BC and 11 healthy tissues from GEO GSE65212 (http://www.ncbi.nlm.nih.gov/geo) as the verification data of the above TCGA and GTEx data. The inclusion criteria of the GEO database are that the number of BC tissues must be more than 100 and must include BC tissues with HER2 + molecular typing. Those that do not meet this requirement will be excluded. The expression levels of SLC31A1 in normal breast tissues and BC tissues were validated using the Human Protein Atlas (THAP) database (https://www.proteinatlas.org/)^14^.

Identification of differentially expressed genes between BC and normal tissue samples.

Raw count data of BC and normal samples from the TCGA and GTEx cohorts were normalized using the Trimmed Mean of M-values method, and comparative analysis was performed using the t-test to determine the differential^15^. Using the formula mean[abs(log2FoldChange)] + 2 × sd[abs(log2FoldChange)], any gene with a false discovery rate (FDR) of < 0.05 and a |log2FoldChange| higher than a cutoff was considered to be a differentially expressed gene. Genes that were consistently upregulated or downregulated in tumor tissues from both cohorts were identified as differentially expressed genes.Data from the GSE65212 cohort were analyzed in a similar manner.

### Cuproptosis-related genes prognostic model

Samples with RNAseq data and clinical information were obtained from the TCGA dataset repository as described above. We performed through multivariate cox regression analysis and then iterative analysis through step function to obtain the prognosis model. We used log rank and univariate Cox regression to compare the difference in survival between two or more groups, and performed timeROC (v 0.4) analysis to determine the accuracy of the discriminant prediction model. The optimal model is selected as the final model.

### Build the nomogram by identifying independent prognostic factors

To find the appropriate nomogram constituents, we applied univariate and multivariate cox regression analysis. Using the R package ‘forestplot’, each variable was shown using the forest plot (R version v4.0.3). The results of the multivariate Cox proportional hazards analysis were used to generate a nomogram to forecast the 2, 3, and 5-year overall recurrence. The nomogram produced a graphical representation of the factors that may be used to forecast the possibility of recurrence for a specific patient, as well as the points related with each risk factor, using the R application ‘rms‘(R version v4.0.3).

### Consistency analysis

Consistency analysis by using ConsensusClusterPlus R package (v1.54.0)^16^, clusterAlg = “hc,” innerLinkage=’ward.D2’, maximum number of clusters is 6, and 80% of the whole sample is drawn 100 times. For clustering heatmaps, use the R software package pheatmap (v1.0.12). Genes with SD > 0.1 are kept in the gene expression heatmap.

### Single-cell analysis

CancerSEA (http://biocc.hrbmu.edu.cn/CancerSEA/), the first specific database targeted at comprehensively deciphering functional states of cancer cells at a single-cell level, was utilized to explore the role of SLC31A1 expression^17^.

### Tumor-infiltrating immune cells and immune checkpoint blockade (ICB) analysis

TIMER (https://cistrome.shinyapps.io/timer/) is a website for analyzing tumor-infiltrating immune cells^18^. To investigate connections between tumor infiltrating immune cells and SLC31A1 expression, the R GSVA package (R version v4.0.3)and TIMER were utilized. SLC31A1 was compared with other biomarkers for the prediction of immune checkpoint inhibitor efficacy by Tumor Immune Dysfunction and Exclusion (TIDE)^19^.

### Protein–protein Interaction network building

The STRING (https://cn.string-db.org/) resulted in a set of 7 SCL31A-binding proteins when the following major settings were established: [“high confidence (0.700)”] is the minimum needed interaction score.

### GO and KEGG enrichment analyses

Gene Ontology (GO) and Kyoto Encyclopedia of Genes and Genomes (KEGG) enrichment analysis for 7 SLC31A1-binding proteins were conducted by using ggplot2 visualization package and the cluster Profiler statistical analysis tool (R version v4.0.3)^19^.

### MiRNA candidate forecast

Numerous target gene prediction algorithms, including TargetScan^20,21^, miRWalk^22^ and miRMap^23^, predicted upstream binding miRNAs of SLC31A1. For the following research, only the projected miRNAs that occurred in all three programs listed above were used. These predicted miRNAs are candidate miRNAs for SLC31A1.

### Candidate lncRNA analysis

StarBase (https://starbase.sysu.edu.cn/) is a database dedicated to RNA research^24^. In BC, starBase was introduced to do miRNA-SLC31A1, lncRNA-miR-195-5p, or lncRNA-SLC31A1 expression correlation analysis. StarBase was used to look at the expression of miR-195-5p in BC and normal controls. In addition, starBase was utilized to identify candidate lncRNAs that may potentially bind to miR-195-5p. Spearman correlation was used to analyze the relationship between SLC31A1 expression and lnRNA or miRNA.

### The relationship between gene expression and drug sensitivity and therapeutic responses

We used the ROC plotter server (www.rocplot.org) to examine the relationships of SLC31A1 transcriptome levels with therapeutic responses in BC patients to determine the effect of SLC31A1 on therapeutic responses^25^. In addition, we used the QUERY module of the Tumor Immune Dysfunction and Exclusion algorithm to assess the impact of genetic and epigenetic alterations in SLC31A1 on dysfunctional T-cell phenotypes^19^.

### Cell culture and transfection

Cell line were from the Chinese Academy of Science (Shanghai, China). The cell line was grown in a sterile and humidified cell culture incubator at 37 °C and 5% CO_2_. MCF-7, BT474, SKBR3 and MDA-MB-231 cell lines was cultured using MEM (Procell, Wuhan, China) containing fetal bovine serum (Invitrogen, Carlsbad, CA) and penicillin (100 U/mL) and streptomycin (100 µg/mL) (P/S, Procell, Wuhan, China) to make up a concentration of 10% FBS and 1% P/S. MCF-10 A was cultured with the following medium: DMEM + 5% HS + 20ng/ml EGF + 0.5 µg/ml hydrocortisone + 10 µg/ml insulin + 1% NEAA + 1% P/S. The serum contains Cucl_2_^3^. To construct SLC31A1 knockdown SKBR3 cell lines. siRNA sequence (sense strand, 5′-CACAAAACUGUUGGGCAAC-3′, antisense, 5′- GUUGCCCAACAGUUUUGUG-3′, GeneChem, Shanghai, China) or siNC (sense strand, 5′-UUCUCCGAACGUGUCACGUTT-3′, antisense, 5′-ACGUGACACGUUCGGAGAATT-3′, GeneChem, Shanghai, China) were transfected into the SKBR3 cell lines using Lipofectamine™ 3000 (Invitrogen) according to the manufacturer’s protocols. After transfection, the SKBR3 cell lines were harvested for further experiments.

### Quantitative real-time polymerase chain reaction (qRT-PCR)

Total RNA was isolated using RNA-easy Isolation Reagent (Vazyme), and cDNA was generated with HiScript^®^ III SuperMix (Vazyme) following the kit instructions. The qRT-PCR was performed in StepOnePlus (Applied Biosystems) equipment with ChamQ™ SYBR^®^ qPCR Master Mix (Vazyme). The specific sequences of the primers used for qRT-PCR are available in Table 1. As an internal reference gene, research utilized glyceraldehyde-3-phosphate dehydrogenase (GAPDH). The 2 − ΔΔct method was used to identify the relative expression levels of SLC31A1.

**Table 1:** Summary of the oligonucleotide primer sequences.

Gene	Forward primer	Reverse primer
GAPDH	GTCTCCTCTGACTTCAACAGCG	ACCACCCTGTTGCTGTAGCCAA
SLC31A1	CCAGGACCAAATGGAACCATCC	ACCACCTGGATGATGTGCAGCA

### Western blot

RIPA buffer was used to thoroughly lyse the cells. SDS-PAGE gels were used to separate the total proteins, which were subsequently transferred to PVDF membranes. Following blocking, membranes were incubated overnight at 4 °C with primary anti-SLC31A1 (1:1000, Abcam, ab129067). The next day, protein bands were seen after appropriately using secondary antibodies. As a control, anti-GAPDH (1:2,000, Abcam, ab8245) was used.

### Cell proliferation assay

Plate clone formation assay: Cell suspensions containing 1,000 cells were then injected into six-well plates for up to 14 days until colonies were evident.The colonies were fixed with 4% paraformaldehyde, stained with 0.5% crystal violet, and counted.

### Cell migration and infiltration assay

Migration and invasion.of cells were assessed by wound healing and Transwell assays. HER2 + cells on 6-well plates were mechanically scraped using a 10 µl pipette to form a line. The cells were washed with PBS to remove debris and cultured in medium containing 1% FBS for 48 h to heal the wound. Transwell permeation analysis was performed using an 8 μm pore size polycarbonate membrane (Corning Inc). The upper chamber was inoculated with serum-free medium, and the medium with 10% FBS was placed in the lower well to serve as a chemical inducer. After 24 h, cells that crossed the membrane were fixed with 4% paraformaldehyde and stained with crystal violet.

### Immunofluorescence

BC tissue was fixed with 4% paraformaldehyde (Sigma-Aldrich), and embedded in paraffin. Anti-SLC31A1(Thermofisher, PA1-16586), and anti-Ki67 (Abcam, ab243878) were used at dilutions of 1:500, and 1:100, respectively. Images were acquired on the fluorescence micro scope(IX71, Olympus).

### In vivo tumor xenografts

This experiment was carried out in accordance with the approval procedure of the Animal Use Committee of Nanjing Medical University. BALB/C nu mice were obtained from the Animal Experiment Center of Nanjing Medical University. BALB/C nu mice(6 weeks old, female) were randomly divided into 2 groups with *n* = 5 in each group. Mice were anesthetized by inhalation anesthesia (2% isoflurane)^26^. The specific cells (1 × 10^6^ cells) were randomly injected subcutaneously into mice. Tumors were sized and their volume estimated over a defined period of time. 28 days after injection, the mice were anesthetized to death, and the mice were subsequently dissected and photographed.

### Euthanasia of BALB/C nu mice

We maintained female 6-week-old BALB/C nu mice under the following conditions: Temperature: 23–25 °C, Relative Humidity: 45–65%, Photoperiod: 12-hour light/12-hour dark cycle. The mice had ad libitum access to food and water. Tumor volume was checked every 7 days after the tumor became palpable. The euthanasia method of BALB/C nu mice complied with the requirements of the American Veterinary Medical Association’s Euthanasia Guidelines 2020 Edition. BALB/C nu mice were euthanized by intraperitoneal injection of sodium pentobarbital (100 mg/kg), combined with local anesthetics and anticonvulsants. Animals were death verified by cervical dislocation^27^.

### Statistical analysis

A t-test was used to assess the differences in variables across groups. For multiple comparisons involving data sets containing 3 or more groups, we used the Kruskal-Wallis Test + Dunn’s test. The connection was evaluated using Spearman correlation analysis. The survival rates were analyzed using the K-M curve. We used Cox regression to find the independent prognostic variables via univariate and multivariate analyses. Statistical significance was considered when p-value < 0.05.

## Result

### Construction of a prognostic signature in cuproptosis-related genes

**Figure 1:**
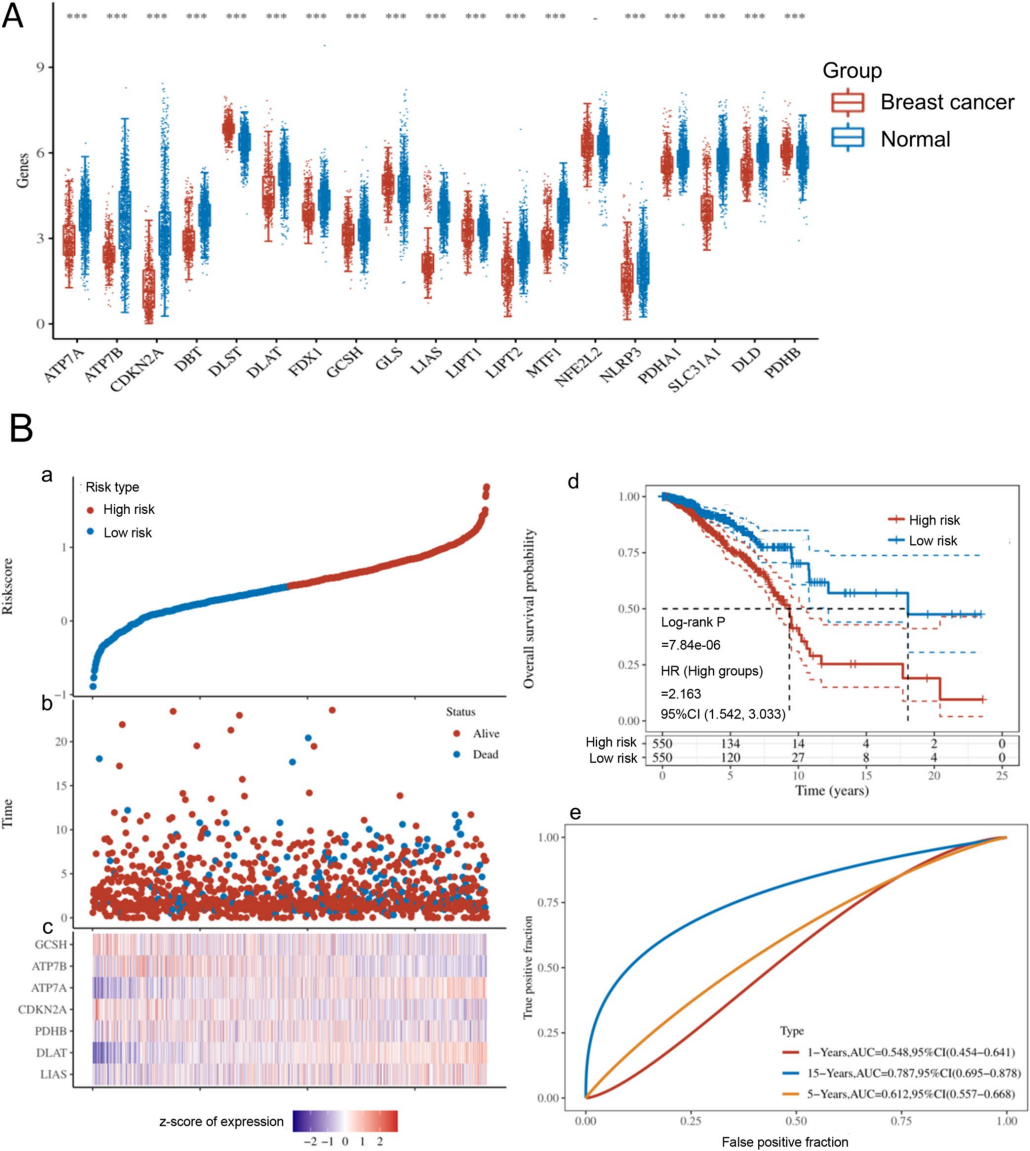
Expression of Cuproptosis-related genes in BC and the construction of a prognostic model. A: The expression distribution of 19 Cuproptosis-related genes in BC tissues and normal breast tissues. The ordinate depicts the expression dispersion of genes, while the abscissa reveals distinct groupings of samples. The Wilcox test was employed to assess statistical differences between the two groups. B: The prognostic model constructed by 19 Cuproptosis-related genes shows its risk score, survival time, and survival status. (a) The risk score is represented by the scatterplot. (b) The scatter plot distribution represents the risk score of different samples corresponding to the survival time and survival status. (c) The heatmap is the gene expression from the signature. (d) Kaplan-Meier survival analysis of the risk model. (e) The ROC curve and AUC of the model. BC, breast cancer; HR, hazard ratio; ROC, receiver operating characteristic; AUC, area under the receiver operating characteristic curve. *p < 0.05, **p < 0.01,***p < 0.001, asterisks (*) stand for significance levels.

Among 19 Cuproptosis-related genes, 18 are differentially expressed in BC and normal breast tissue (Fig. 1A). The scatter plot risk and score distribution plot indicated that the low-risk subgroup had lower risk scores and better survival times than that of the high-risk subgroup ( Fig. 1B, a-b ) and the expression levels of some Cuproptosis-related genes were shown in the heatmap (Fig. 1B, c). K-M survival curves showed that the outcomes of BC cases in the low-risk subgroup were better than those with high-risk (*p* = 7.84e-06) (Fig. 1B, d). ROC curves presented the highest AUC value of 0.79 (Fig. 1B, e) in predicting 15-year survival, demonstrating that the prognostic signature had a good promising ability.

### Construction and comparison of three clusters of BC by cuproptosis-related genes

We divided the 1097 BC samples into three clusters based on the expression similarity of cuproptosis-related genes (Supplementary Fig. 1A-B). 776 cases in C1, 177 in C2, and 144 in C3 were among them. Consensus clustering matrix for k = 3 (Supplementary Fig. 1A). The heatmap of gene expression associated with cuproptosis in three subgroups (Supplementary Fig. 1C). We subsequently investigated the clinical features. The significance of the BC cluster was confirmed by the fact that a notably higher number of patients in the C2 group than in the other two groups underwent chemotherapy (Supplementary Fig. 1D).The prognostic values of SLC31A1 in BC.

Following the aforementioned analysis, we conducted the following analysis in order to identify the most important Cuproptosis-related genes in BC for further study.

**Figure 2:**
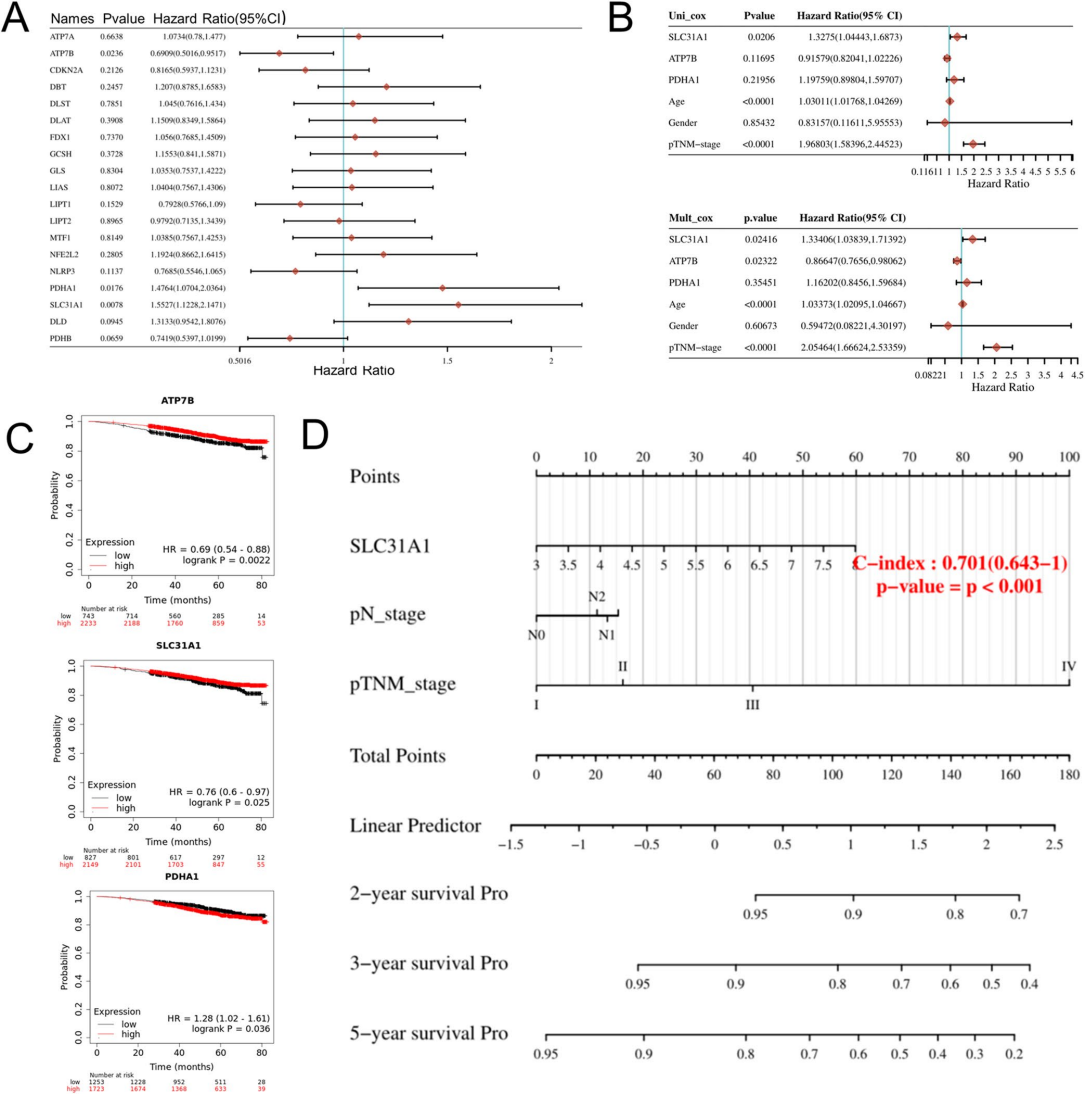
SLC31A1 serves as an independent prognostic factor of BC. (**A**) In the forest plot, KM survival analysis Cuproptosis-related genes of BC, only SLC31A1, ATP7B and PDHA1 have survival differences. (**B**) Analysis of P values by univariate and multivariate Cox regression showed that only SLC31A1 could serve as an independent prognostic factor of BC. (**C**) KM survival curve analysis with survival difference SLC31A1, ATP7B and PDHA1. (**D**) Nomogram based on SLC31A1 can predict the 2-year, 3-year and 5-year overall survival of BC patients. KM, Kaplan–Meier. BC, breast cancer.

First, after the Kaplan–Meier (KM) survival analysis, only SLC31A1, ATP7B, and PDHA1 have survival differences among Cuproptosis-related genes (Fig. 2A and C) in BC. However, univariate and multivariate cox regression analysis of P values revealed that only SLC31A1 (*P* < 0.05) could be applied as an independent predictive factor (Fig. 2B). Second, the 2-year, 3-year, and 5-year overall survival of BC patients may be predicted using a nomogram based on SLC31A1 (Fig. 2D). Finally, SLC31A1 was found to be predominantly involved in regulating proliferation, DNA repair, and apoptosis in single cells, particularly in BC (Supplementary Fig. 2).

### The clinical significance of SLC31A1 in BC

**Figure 3:**
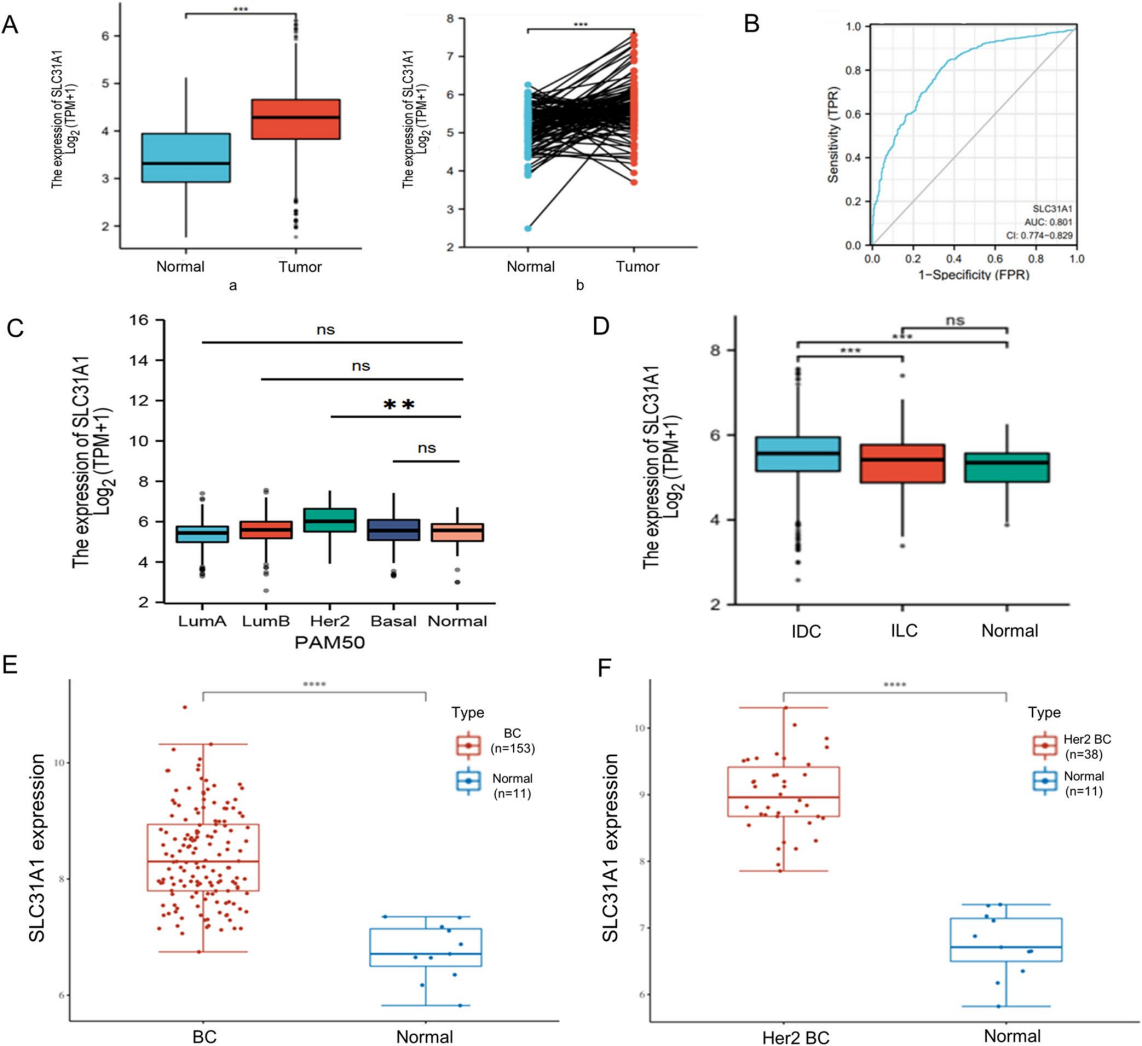
Clinical significance of SLC31A1 expression in BC. (**A**) Compared with normal breast tissue (Mean expression: 3.425), SLC31A1 is highly expressed in BC (Mean expression: 4.234). a.unpaired analysis. b. right, paired analysis. (**B**) In predicting normal breast tissue and BC outcomes, the predictive ability of SLC31A1 had high accuracy (AUC = 0.801, CI = 0.774–0.829). (**C**) Expression of SLC31A1 in luminal A, luminal B, HER2+, and basal BC compared to normal breast tissue. (**D**) Compared with normal breast tissue, the expression of SLC31A1 in IDC and ICL. IDC, Infiltrating Ductal Carcinoma; ILC, Infiltrating Lobular Carcinoma. In the expression data GSE 65,212, SLC31A1 is highly expressed in BC (**E**) and Her 2 + enriched BC (**F**). BC, breast cancer; AUC, area under the receiver operating characteristic curve; CI, confidence intervals; IDC, Infiltrating Ductal Carcinoma; ILC, Infiltrating Lobular Carcinoma. *p < 0.05, **p < 0.01,***p < 0.001.

**Figure 4:**
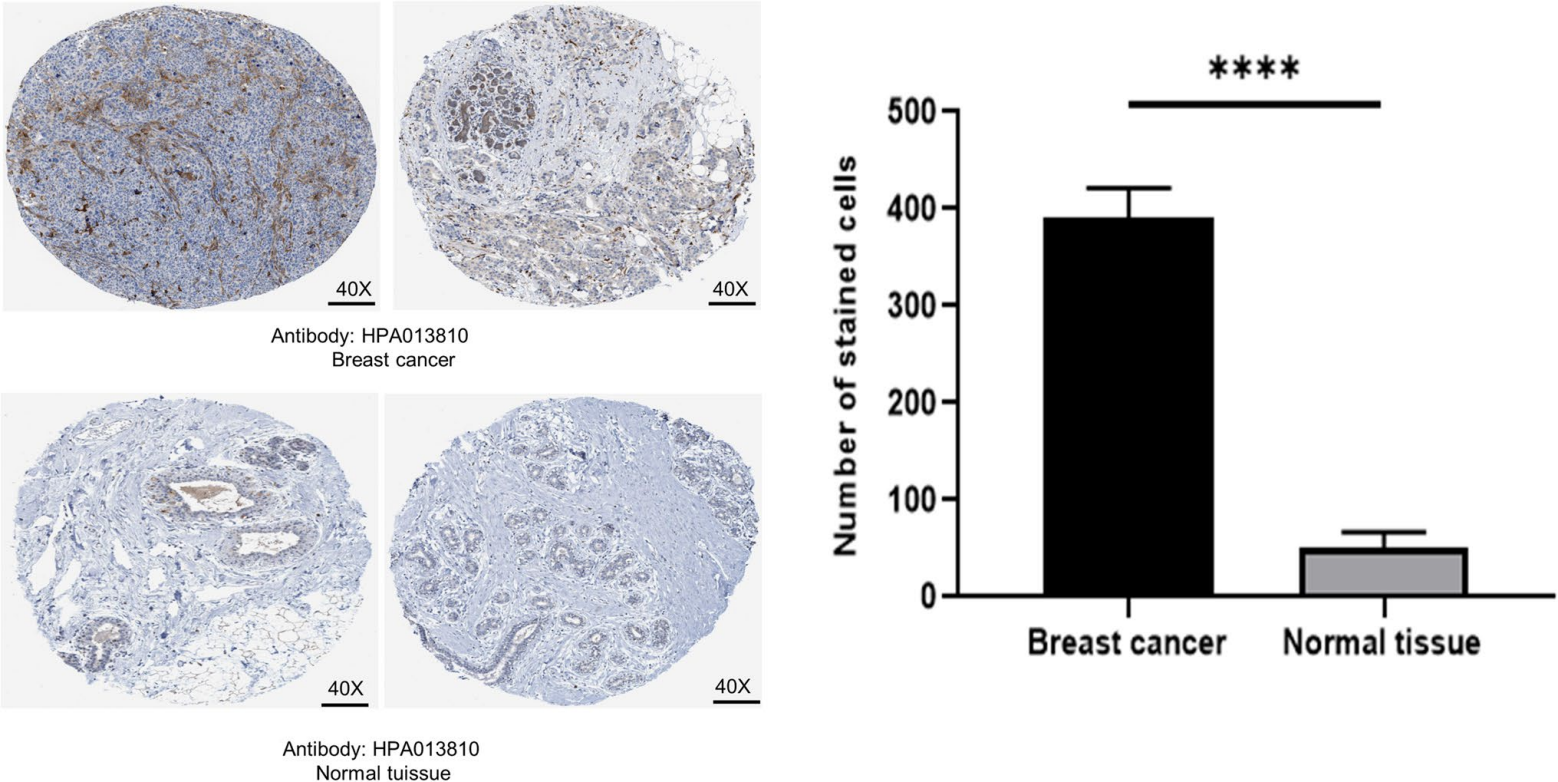
Clinical significance of SLC31A1 expression in BC. SLC31A1 protein expression in BC specimens and non-cancerous breast tissues through THPA website analysis. BC, breast cancer; THPA, The Human Protein Atlas. *p < 0.05, **p < 0.01,***p < 0.001.

In the TCGA and GTEx data, SLC31A1 is highly expressed in BC when compared with normal breast tissue in both paired and unpaired analyses (Fig. 3A, a-b). SLC31A1 demonstrated a high degree of accuracy in predicting normal breast tissue or BC (Fig. 3B, AUC = 0.801, CI = 0.774–0.829). Specifically, in molecular typing, the expression of SLC31A1 is higher in HER2-enriched BC when compared with normal breast tissue(Figure 3C). In pathological classification, the expression of SLC31A1 is higher in Infiltrating Ductal Carcinoma than Infiltrating Lobular Carcinoma and normal breast tissue (Fig. 3D). In the independent gene expression data GSE 65212, we also found that SLC31A1 is highly expressed in BC (Fig. 3E) and Her 2 + enriched BC (Fig. 3F). This is consistent with our results in the TCGA and GTEx data. Moreover, SLC31A1 is higher in ER negative, PR negative and HER2 positive than ER positive, PR positive and HER2 negative (Supplementary Fig. 3A-C). By immunohistochemical analysis of The Human Protein Atlas (THPA) website, it could also be found that SLC31A1 was highly expressed in BC compared to non-cancerous breast tissue (Fig. 4). In the Kaplan-Meier curves, SLC31A1 is a negative factor for overall survival (OS)(Fig. 2C).

### Association of SLC31A1 expression with immune infiltration levels in BC

**Figure 5:**
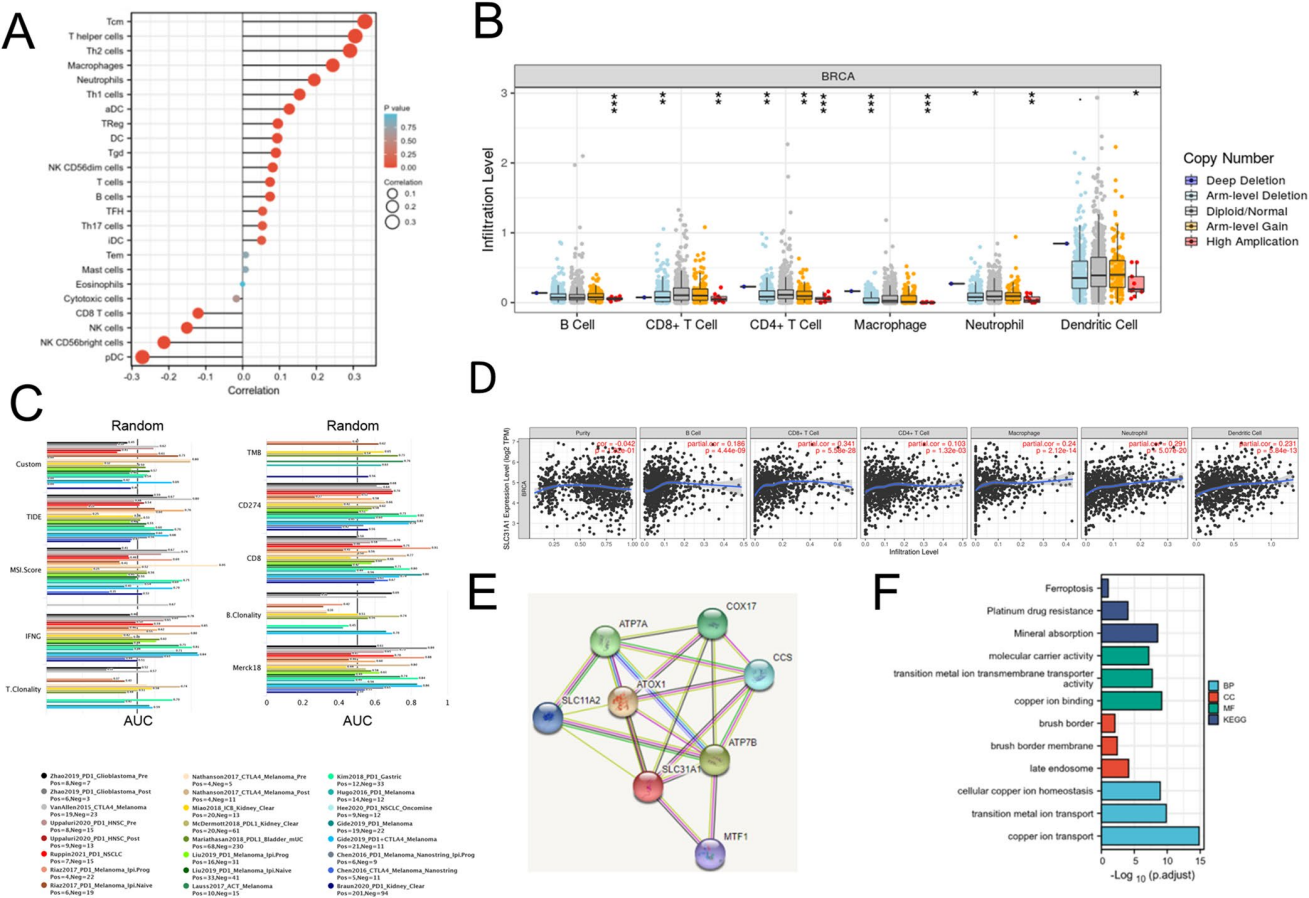
The potential relationship between SLC31A1 expression and immune infiltration levels in BC. (**A**) Tcm, T helper cells, Th2 cells had the strongest positive link with SLC31A1, while pDC, NK CD56bright cells, and NK cells showed the strongest negative correlation. (**B**) In BC, there exists a connection between SLC31A1 gene copy number and immune cell infiltration levels. (**C**) Bar plot of SLC31A1 biomarker relevancy in ICB sub-cohorts when compared with standardized cancer immune evasion biomarkers. The AUC was adopted for assessing the test biomarkers’ prediction abilities on the ICB response status. (**D**) Correlation of SLC31A1 expression with immune infiltration level in BC. (**E**) SLC31A1 shows a strong correlation (High confidence = 0.07) of genes for PPI network building. (**F**) The GO and KEGG analysis of SLC31A1 highly associated genes. BC, breast cancer; ICB, immune checkpoint blockade; AUC, area under the receiver operating characteristic curve; PPI, protein-protein interaction. GO, Gene Ontology; KEGG, Kyoto Encyclopedia of Genes and Genomes(www.kegg.jp/kegg/kegg1.html). aDC (activated DC); iDC (immature DC); pDC (Plasmacytoid DC); Tcm(T central memory); Tem (T effector memory); Tgd (T gamma delta); Tfh (T follicular helper).

The following investigation was performed to observe whether there existed a link between SLC31A1 expression and immune infiltration levels in BC. First, TIMER was used to investigate the relationships between SLC31A1 expression and other immune infiltration indicators. The obtained findings revealed a significant relationship between SLC31A1 expression and all of the Monocyte, TAM, and M2 macrophage marker sets, as well as a significant relationship between SLC31A1 expression and the majority of T cell (general), M1 macrophage, Neutrophils, Natural killer cell, Dendritic cell, and Th2 cell marker sets (Table 2). Full information is in the Supplementary table. Second, we found that Tcm, T helper cells, and Th2 cells had the strongest positive link with SLC31A1, while pDC, NK CD56bright cells, and NK cells have the strongest negative correlation (Fig. 5A). The TIMER module’s “Somatic copy number alterations (SCNA)” analysis revealed that numerous immune cell infiltration levels appeared to be linked to changed SLC31A1 gene copy numbers (Fig. 5B). According to the SCNA module, the arm-level deletion of SLC31A1 was substantially related to immune cell infiltration levels in BC. SLC31A1 expression has a substantial link with tumor purity, and it is markedly positively correlated with infiltrating levels of B lymphocytes, CD8 + T cells, CD4 + T cells, macrophages, neutrophils, and dendritic cells in BC, based on the “Gene of TIMER” module study (Fig. 5D). Finally, SLC31A1’s biomarker significance was assessed by comparing that to standard biomarkers that could predict ICB sub-cohort response outcomes. In 11 of the 23 ICB subcohorts, SLC31A1 exhibited an area under the receiver operating characteristic curve (AUC) of > 0.5 (Fig. 5C). TMB, T.Clonality and B. Clonality demonstrated stronger predictive value than SLC31A1, with AUC values of > 0.5 in eight, seven, and six ICB sub-cohorts. (Fig. 5C).

**Table 2:** Correlation analysis between SLC31A1 and related genes and markers of immune cells in bc by TIMER. (The following statistical test methods all use Spearman’s rho value).

	Gene markers	BC			
		None		Purity	
		Correlation	p	Correlation	p
CD8 + T cell	CD8A	0.113014782	0.000173	0.114734494	0.00028721
T cell (general)	CD3D	0.045382095	0.132525	0.050622614	0.11052529
	CD3E	0.080420961	0.007618	0.084116189	0.0079379
	CD2	0.132651739	1.01E-05	0.134336186	2.12E-05
Monocyte	CD86	0.243706625	2.45E-16	0.244397354	5.35E-15
	CD115 (CSF1R)	0.172854563	7.93E-09	0.164909528	1.68E-07
TAM	CCL2	0.097665687	0.001182	0.103029944	0.00113586
	CD68	0.245433112	1.49E-16	0.244975278	4.60E-15
	IL10	0.238154676	1.19E-15	0.23929394	2.01E-14
M1 macrophage	INOS (NOS2)	0.096619579	0.001335	0.096219854	0.0023787
	COX2 (PTGS2)	0.123260402	4.15E-05	0.12836951	4.89E-05
M2 macrophage	CD163	0.304902114	4.26E-25	0.30732745	3.30E-23
	VSIG4	0.200770923	1.82E-11	0.197455747	3.33E-10
	MS4A4A	0.256538792	5.44E-18	0.25809753	1.32E-16
Neutrophils	CD11B (ITGAM)	0.252212802	2.01E-17	0.236764422	3.82E-14
	CCR7	0.078433156	0.009258	0.087413353	0.00579518
Natural killer cell	KIR2DL1	0.101464351	0.000752	0.11361554	0.00032945
	KIR2DL3	0.11937422	7.22E-05	0.119313104	0.00016174
	KIR2DL4	0.08898746	0.003138	0.091387412	0.00391283
	KIR3DL1	0.088118203	0.003445	0.08819079	0.00537285
	KIR3DL2	0.101575434	0.000741	0.105529982	0.00085629
	KIR2DS4	-0.380028495	0.000551	-0.345055408	0.00260562
Dendritic cell	HLA-DPB1	-0.282935735	0.01175	-0.323035913	0.00518271
	HLA-DRA	0.220164534	1.53E-13	0.213764467	9.54E-12
	HLA-DPA1	0.171455903	1.05E-08	0.165627756	1.49E-07
	BDCA-4 (NRP1)	0.316076332	6.06E-27	0.311878143	6.92E-24
	CD11c (ITGAX)	0.174870818	5.28E-09	0.173186308	3.85E-08
Th2	GATA3	0.478042843	1.08E-05	0.454098482	5.83E-05
	STAT5A	-0.215287244	0.056881	-0.264272492	0.02316128
	IL13	0.367704783	0.000857	0.377435062	0.00091621

### Research of the PPI network, GO, and KEGG enrichment

Using the STRING database, we identified 7 SLC31A1 targeted binding proteins (Fig. 5E). Then, by performing GO enrichment analysis (Fig. 5F), we discovered that copper ion transport, transition metal ion transport, and cellular copper ion homeostasis were among the key biological processes (BP). In addition, late endosomes, brush border membranes, and brush border were all high in cellular component (CC). Copper ion binding, transition metal ion transmembrane, transporter activity, and molecular carrier activity were all engaged in the molecular function (MF). Besides, mineral absorption, platinum drug resistance, and ferroptosis were the most common KEGG pathway enrichments (Fig. 5F).

### SLC31A1 upstream miRNA prediction and analysis

**Figure 6:**
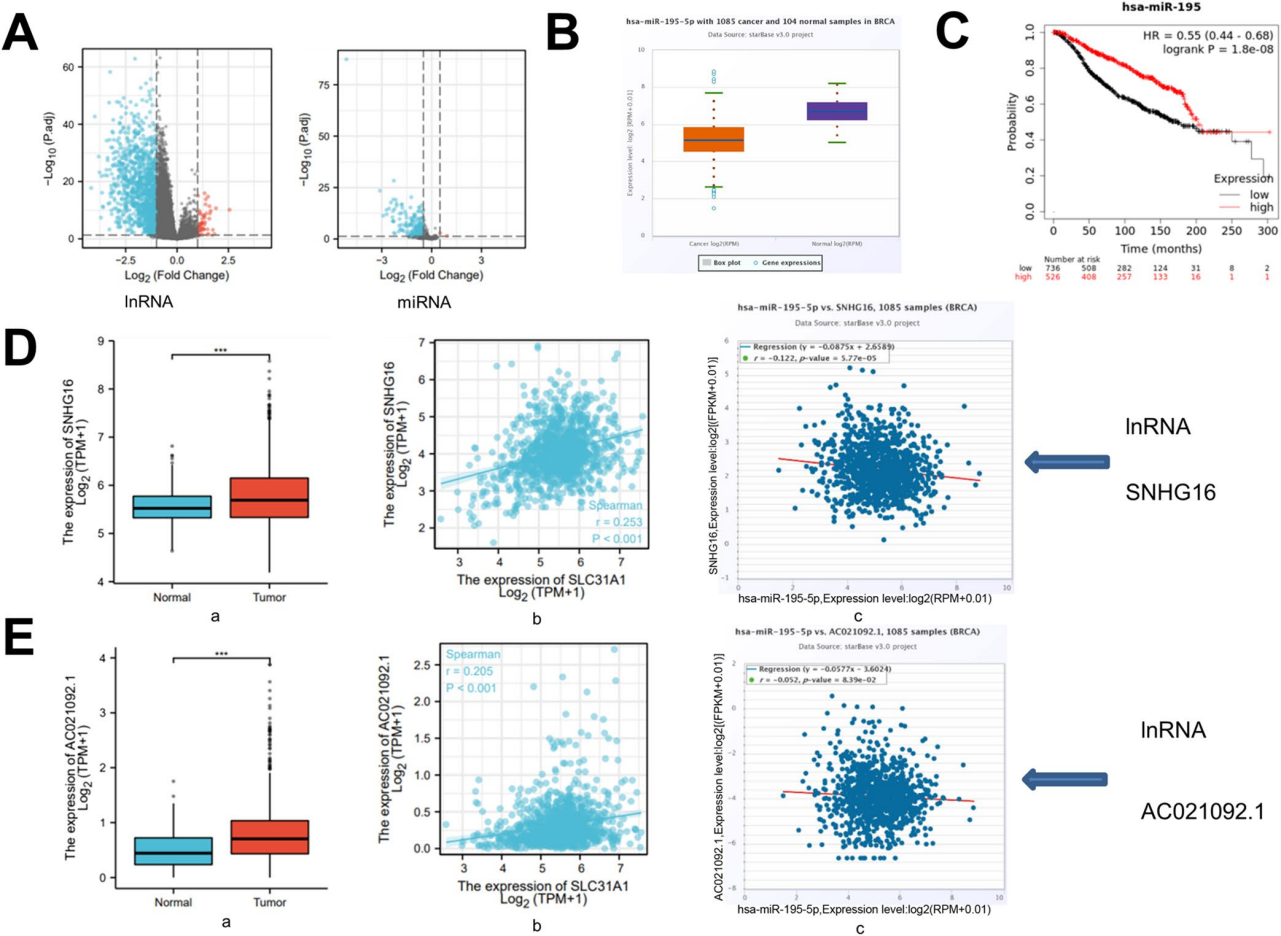
Identification of upstream miRNAs and lnRNAs regulating SLC31A1. (**A**) The volcano plots describe 7021 DElncRNAs (|log2fold change| > 0.5 and adjusted *p* value < 0.05), 319 DEmiRNAs (|log2fold change| > 0.5 and adjusted *p* value < 0.05) in association with SLC31A1. Red represents upregulated genes and blue indicates downregulated genes. (**B**) Compared with normal breast tissue, hsa-miR-195-5p is highly expressed in BC. (**C**) High expression of hsa-miR-195-5p was shown to be a detrimental factor for OS by Kaplan-Meier methods. (**D**) Expression analysis and correlation analysis for upstream lncRNAs (SNHG16) of miR-195-5p in BC. Compared with normal breast tissue, SNHG16 is highly expressed in BC (a); SNHG16 is positively correlated with SLC31A1 in BC (b); SNHG16 is negatively correlated with hsa-miR-195-5p in BC (c). (**E**) Expression analysis and correlation analysis for upstream lncRNAs (AC021092.1) of miR-195-5p in BC. Compared with normal breast tissue, AC021092.1 is highly expressed in BC (a); AC021092.1 is positively correlated with SLC31A1 in BC (b); AC021092.1 is negatively correlated with hsa-miR-195-5p in BC (c). BC, breast cancer; DE, differentially expressed.

The role of ncRNAs in gene regulation has long been recognized. The volcano plots describe 7021 differentially expressed (DE) lncRNAs and 319 DEmiRNAs associated with SLC31A1 (Fig. 6A). To ascertain whether ncRNAs influenced SLC31A1, we started by looking for upstream miRNAs that may bind to SLC31A1. Then, we discovered 319 of them. Based on miRNA’s action method in controlling target gene expression, there should be a negative correlation between miRNA and SLC31A1. Subsequently, research on expression connection was conducted. Among them, a total of 124 miRNAs were negatively correlated with SLC31A1. The negative link between SLC31A1 and miR-628-3p and miR-195-5p was highest in BC. Only the expression of miR-195-5p differed between BC and normal breast tissues. Finally, the expression of miR-195-5p in BC, as well as its prognostic value, were identified. MiR-195-5p was significantly downregulated in BC, as seen in Fig. 6B and C, and its overexpression was associated with patients’ prognosis. All of these data indicate miR-195-5p as the most promising SLC31A1 regulating miRNA in BC.

### Forecasting and evaluation of SNHG16 and AC021092.1 upstream lncRNAs

According to the competing endogenous RNA (ceRNA) theory, with the starBase database, the upstream lncRNAs of miR-195-5p were predicted. lncRNA will increase mRNA production by competitively binding to conserved miRNAs. As a result, the relationship between lncRNA and miRNA should be negative, whereas the relationship between lncRNA and mRNA should be positive. MiR-195-5p was linked to 42 different lnRNAs. GEPIA was then used to quantify the expression levels of these lncRNAs in BC. Among all 42 lncRNAs, 6 lncRNAs were significantly upregulated and had prognostic values in BC compared with normal controls. SNHG16, LINC00662 and AC021092.1 are positively correlated with SLC31A1. The lnRNAs negatively correlated with only hsa-miR-195-5p were SNHG16 (Spearman correlation coefficient, *r* = − 0.122) and AC021092.1 (Spearman correlation coefficient, *r* = − 0.052), of which SNHG16 had the strongest negative correlation (Fig. 6D-c and E-c). As suggested in Fig. 6D-E, compared with normal breast tissue, SNHG16 (Fig. 6D-a) and AC021092.1 (Fig. 6E-a) were highly expressed in BC and had a significant positive correlation with SLC31A1 (Fig. 6D-b and E-b).

Based on expression and correlation analyses, SNHG16 may be the most potential upstream lncRNAs of the miR-139-5p/SLC31A1 axis in BC.

### In BC, SLC31A1 expression is related to treatment responses

**Figure 7:**
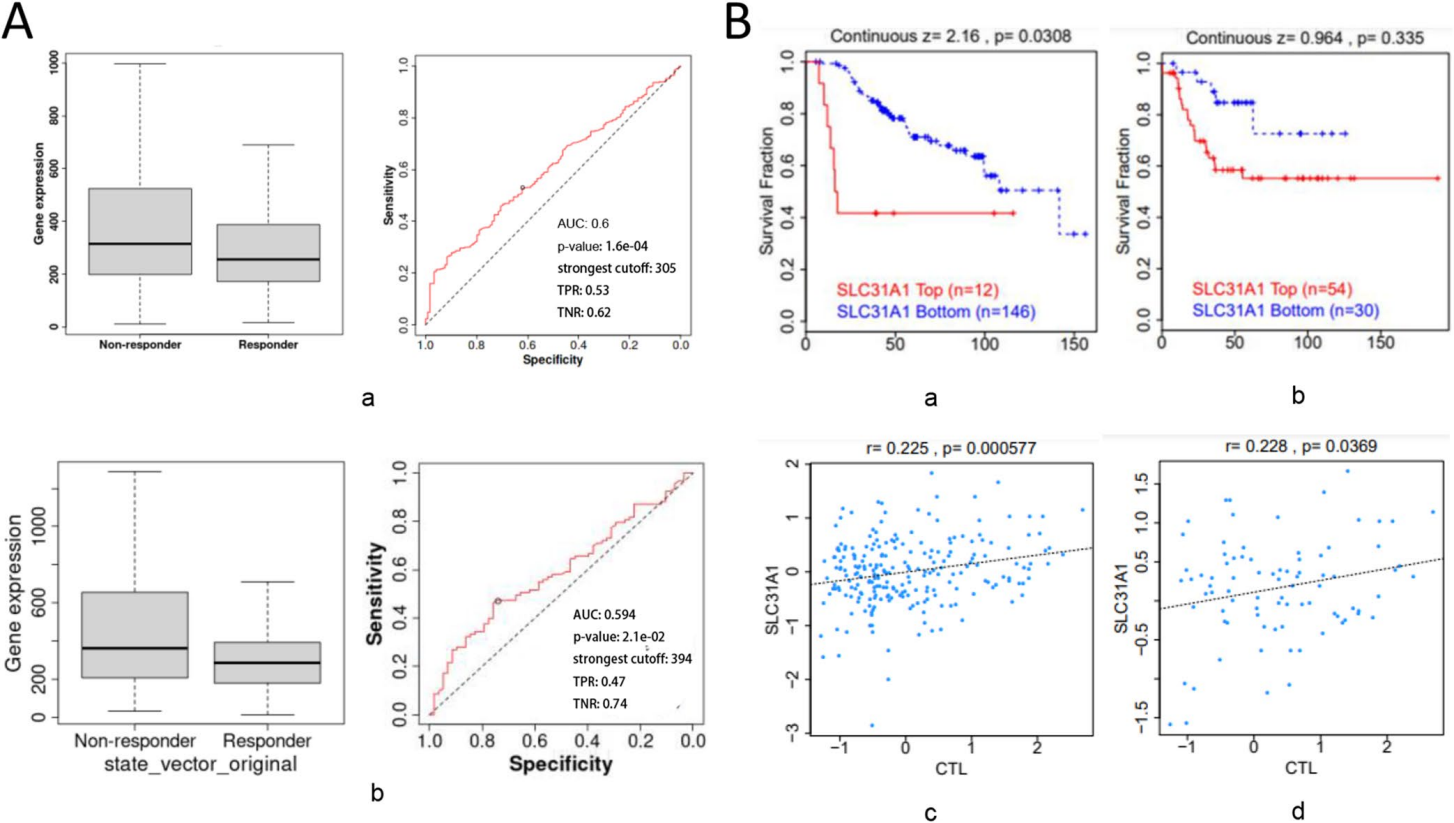
In BC, SLC31A1 expression is related to treatment responses. (**A**) ROC curve depiction illustrating the relationship between SLC31A1 expression and chemotherapy (a) or trastuzumabin (b) responses in BC. (**B**) Survival ratios as a metric of immunotherapeutic response (immune checkpoint blockade) between BC (a) and triple-negative BC (b) with high and low SLC31A1 expression levels (upper panel). The connection between SLC31A1 expression and cytotoxic T-cell level (CTL) in various cohorts is shown in the lower panel. Only BC (c) and its molecular subtype triple negative BC (d) were statistically significant differences. BC, breast cancer; ROC, receiver operating characteristic; CTL, cytotoxic T-cell level.

The impact of SLC31A1 expression on chemotherapy responses in clinical BC was investigated. Patients with high SLC31A1 expression were shown to be resistant to chemotherapy (Fig. 7A-a). BC patients with high SLC31A1 expression are also resistant to Trastuzumab, the target drug of Anti-HER2 therapy (Fig. 7A-b). Although *P* = 0.052, with the increase in sample size, the P value of this result may be statistically significant.

Furthermore, we discovered that lower SLC31A1 expression levels were related to the therapeutic advantages of ICB treatment (PD-1 or PD-L1) in BC and triple negative BC. We had longer survival durations than high SLC31A1 expression levels (Fig. 7B, a-b). Although the same conclusion could not be drawn in triple-negative BC (*P* = 0.335), the trends shown in Fig. 7B-b appear to reflect the same clinical benefit.

SLC31A1 expression levels in BC and triple negative BC were favorably related to cytotoxic T-cell levels (CTL), implying a relationship with T cell promotion (Fig. 7B, c-d).

### Knockdown of SLC31A1 inhibits BC growth

To further elucidate the clear independent role of SLC31A1 in BC, MCF-7 (Luminal A BC cells), BT474 (Luminal B BC cells), SKBR3 (Her2 + enriched BC cells), MDA-MB-231 (Basal like BC cells) were selected for in vitro experiment. The expression of SLC31A1 in Her2-enriched cells was higher than that of the other three cell lines. Therefore, Her2-enriched cells were selected for the follow-up in vitro and in vivo experiments.

**Figure 8:**
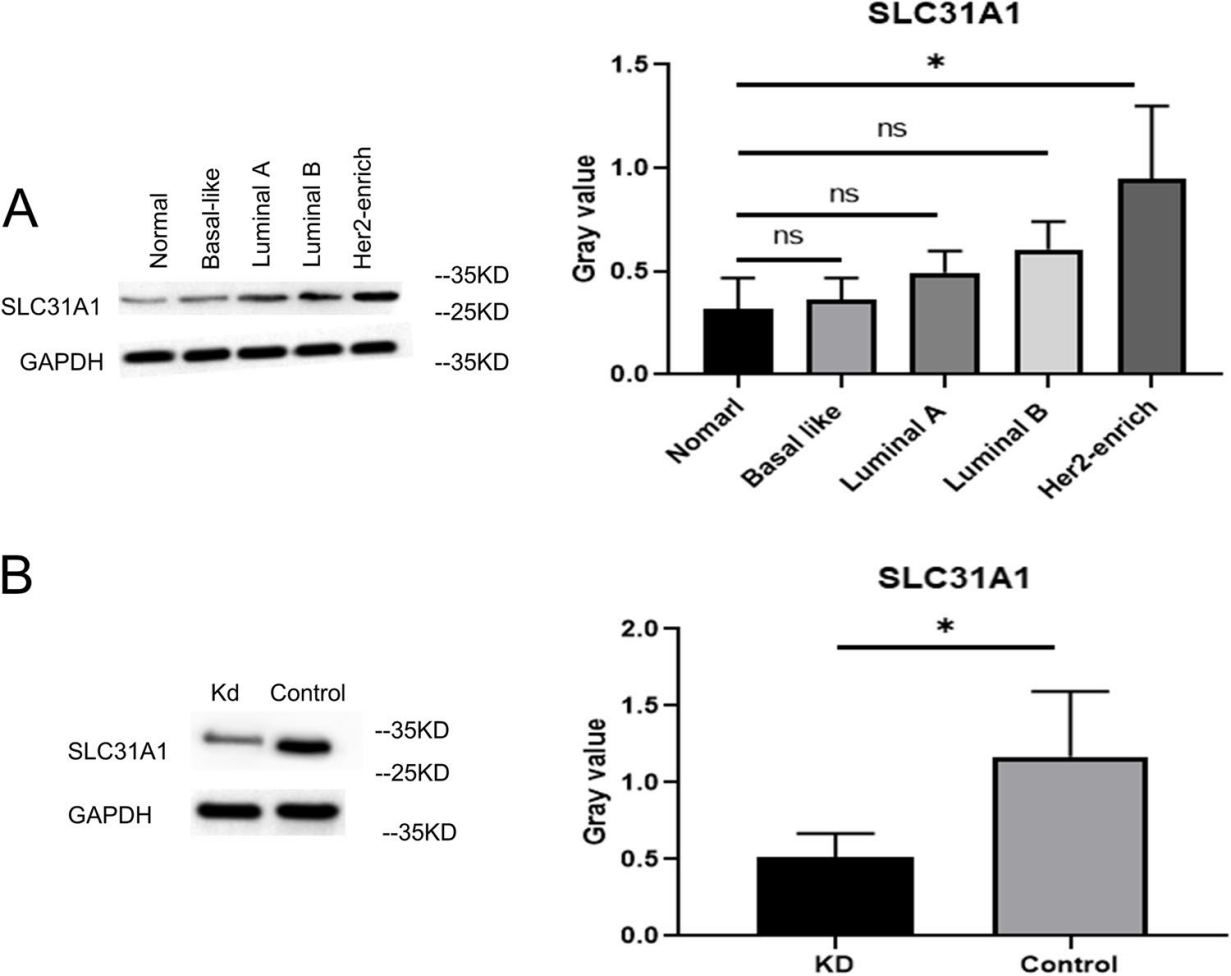
Expression of SLC31A1 in BC cell lines. (**A**) Expression of SLC31A1 in normal breast cells, Luminal A, Luminal B, Basal-like and Her2 + enriched BC cell lines. (**B**) Successful establishment of SLC31A1 knockdown Her2 + enriched BC cells. BC, breast cancer.

**Figure 9:**
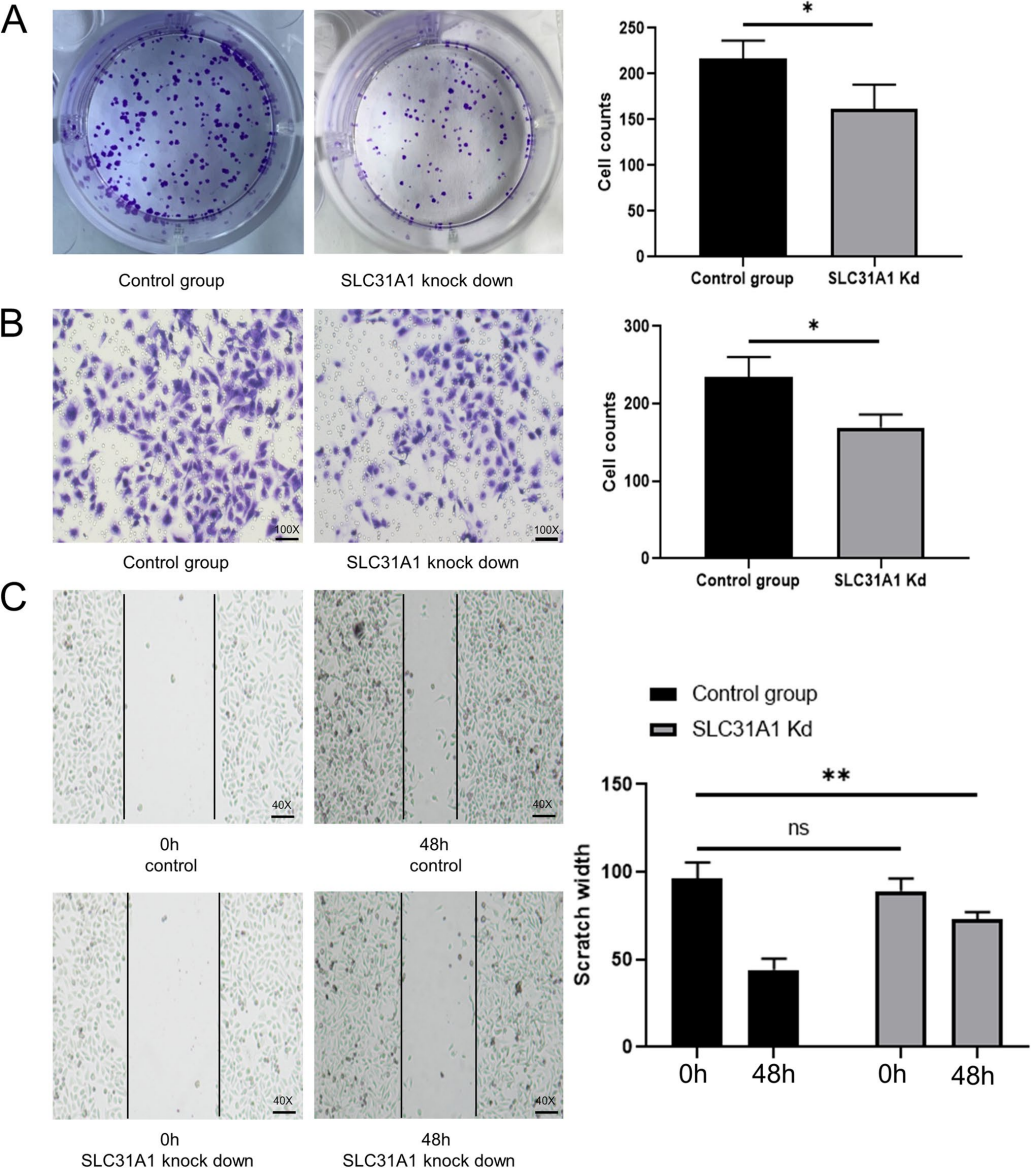
Silencing SLC31A1 dramatically inhibits the proliferation and migration of Her2 + enriched BC cells in vitro. (**A**) Plate clone formation assay showed that the proliferation of Her2 + enriched BC cell lines was attenuated after SLC31A1 knockdown. B, C. Transwell infiltration (**B**) and Wound healing assay (**C**) experiment indicate that SLC31A1 knockdown inhibits the migration and invasion of Her2 + enriched BC cells. BC, breast cancer.

We first verified the expression of SLC31A1 in normal breast cells, Luminal A, Luminal B, Basal-like and Her2 + enriched BC cell line (Fig. 8A). Then, we successfully constructed SLC31A1 knockdown Her2 + enriched BC cells (Fig. 8B). The results of the plate clone formation assay showed that knockdown of SLC31A1 reduced the proliferation of Her2 + enriched BC cells (Fig. 9A). Both the transwell assays (Fig. 9B) and wound-healing assays (Fig. 9C) demonstrated reduced Her2 + enriched BC cells migration after knockdown of SLC31A1.

**Figure 10:**
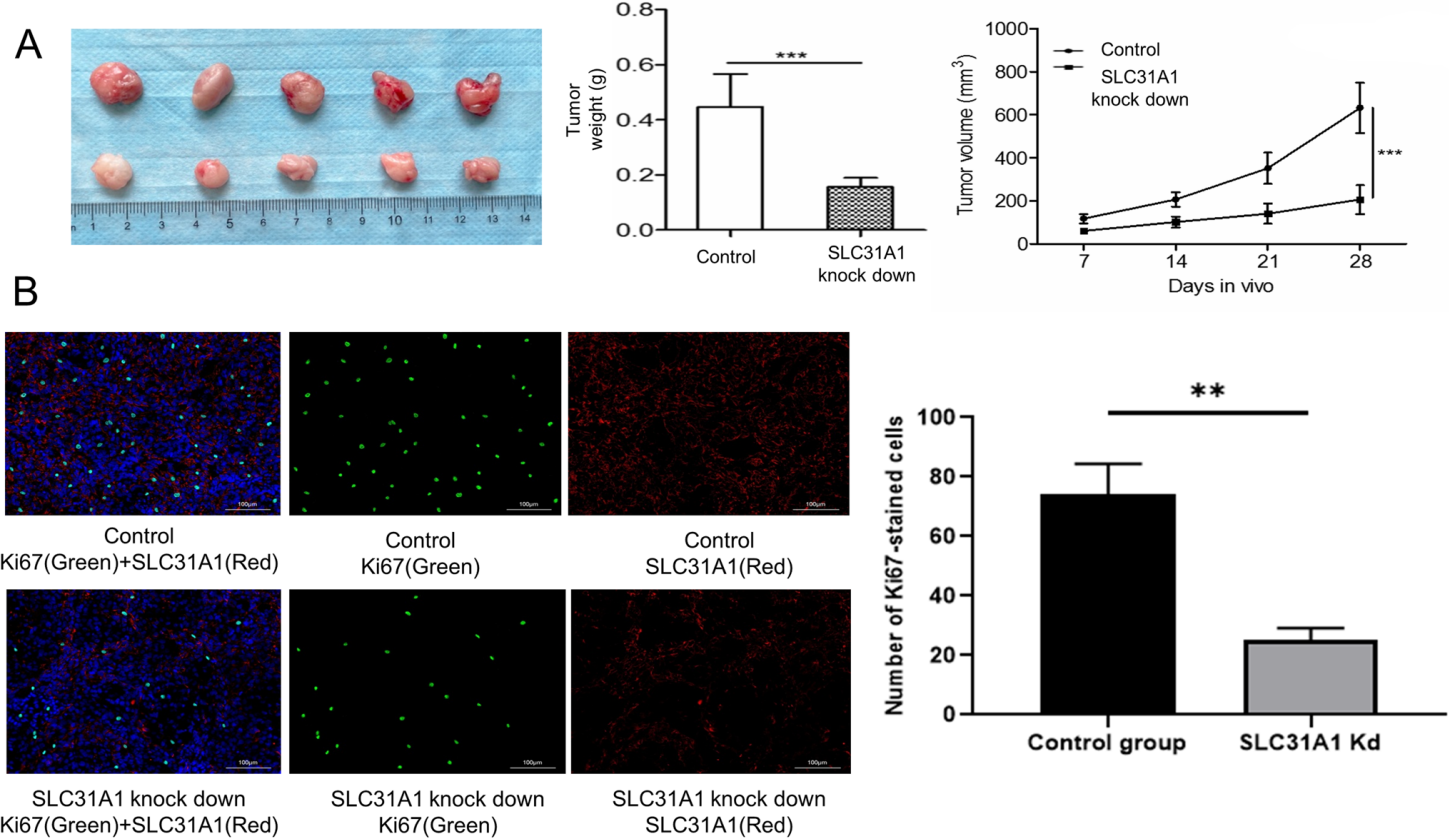
SiSLC31A1 inhibits the proliferation of Her2 + enriched BC cells in vivo. (**A**) In subcutaneous tumorigenesis experiments with BALB/C nu mice, Her2 + enriched BC masses gradually shrunk in vivo after SLC31A1 knockdown. (**B**) Immunofluorescence results showed that the proliferation of Her2 + enriched BC was attenuated in the SLC31A1 knockdown group compared to the control group. Ki67 positivity is indicated by green fluorescence. SLC31A1 positivity is indicated by red fluorescence. BC, breast cancer.

6-week-old BALB/C nu mice were experimentally studied with subcutaneous xenografts. Afterwards, SLC31A1-knockdown tumors progressively shrank in vivo (Fig. 10A), showing that SLC31A1, a tumor-promoting factor, significantly downregulated cell proliferation after its knockdown in Her2 + enriched BC cells.

The immunofluorescence of Ki67 was used to assess the proliferation of Her2 + enriched BC. The proportion of Ki67-positive cells in Her2 + enriched BC in 28-day lesions was considerably more remarkable than the knockdown of SLC31A1 Her2 + enriched BC in BALB/c nu mice (Fig. 10B).

Therefore, we confirmed that upregulated SLC31A1 could facilitate the progression of Her2 + enriched BC cells both in vitro and in vivo.

## Discussion

BC is still renowned for its significant incidence and intricacy today^28^. The discovery of the molecular mechanism of BC carcinogenesis could lead to the development of viable therapy targets or the identification of promising prognostic markers. According to a published study, copper toxicity is caused by a process distinct from other known mechanisms of regulated cell death^3^.

Besides, copper ionophores^29–31^ and copper chelators^32,33^ have been suggested as anticancer agents, thus providing new insight and directions for us to find new therapeutic targets for BC. The link between Cuproptosis-related genes and BC is a critically needed area of research.

It has been reported that a prognostic risk signature built by ferroptosis-related genes could predict the prognosis of BC or other cancer patients precisely^34,35^. However, it is unclear whether a prognostic signature established by Cuproptosis-related genes could be used to predict the prognosis of BC. Copper-induced cell death may play a role in BC, motivating different trajectories of cell biology in BC.

In our study, we first constructed a prognostic signature with the 19 Cuproptosis-related genes and divided BC patients into different subgroups using TCGA and GTEx data. We discovered that high-risk patients had a lower life expectancy than low-risk individuals. To explore which Cuproptosis-related genes have a role, we conducted an in-depth exploration.

Solute carrier (SLC) family members are essential in transporting amino acids across membranes^36^. SLC act as tumor suppressors or promoters, affecting the cancer methylome, tumor growth, cancer cell metabolism, immune escape, and chemoresistance^37–39^. SLC31A1, a member of the SLC family, is essential to our research. We identified only SLC31A1 among 19 Cuproptosis-related genes as an independent prognostic factor for BC using univariate and multivariate Cox regression. Built on SLC31A1, we created a Nomogram plot that can predict the OS of BC patients. It has been reported that SLC31A1 affects the development of tumors, such as BC and pancreatic cancer, which depend on copper levels^40^.This research, combined with our analytic findings, demonstrated SLC31A1’s oncogenic function in BC. Furthermore, we found that SLC31A1 was not only highly expressed in BC, but also had a high accuracy in its predictive power for BC. Regarding more specific typing, SLC31A1 is highly expressed in BC of HER2 + or Infiltrating Ductal Carcinoma (IDC). Other critical clinical implications include that SLC31A1 negatively correlates with OS. These studies showed that SLC31A1, as a biomarker, can predict BC deeply to the level of molecular typing and pathological typing. It may have important implications for the clinical decision-making of SLC31A1-based BC therapy, mainly targeted therapy^41^.

Tumor-associated immune cells have recently received much focus. SLC31A1 was found to be inversely related to tumor-antagonizing immune cells^42^, such as NK cells, in our research. Plasmacytoid Eosinophils (pDC) are increased in tumors. They are usually associated with a good prognosis^43^. In contrast, pDC was down-regulated with up-regulation of SLC31A1. This reflects that SLC31A1 may downregulate the infiltrating levels of NK cells and pDC to promote the development of BC.

The presence of SLC31A1 in BC is consistent with skewing of the T central memory cell (Tcm) phenotypes, which are associated with anti-tumor response to ICB^44^. Antibodies against PD-1 or PDL-1 are effectively treat a wide range of malignancies and improve prognoses^45^. According to our findings, BC and triple-negative BC with low SLC31A1 expression reaped more incredible clinical benefits from ICB therapy (PD-1 or PD-L1).

Chemotherapy drugs inhibit tumor growth by preventing the production of nucleotides, blocking proteins and cell structures necessary for cancer cells to replicate^46^. All patients with metastatic BC will receive chemotherapy alone or in combination^47^. We found that increased SLC31A1 expression levels were positively correlated with CTLs. CTLs may be reliable markers for predicting pathological complete response rates after neoadjuvant chemotherapy due to their antitumor cytotoxic activity^48^. However, BC patients will develop chemotherapy resistance after chemotherapy^49^. Interestingly, our study further found that high SLC31A1 expression was resistant to chemotherapy and Trastuzumab, which suggests that SLC31A1 provides a therapeutic marker for addressing chemotherapy and targeted drug resistance in BC.The reason behind it deserves further study.

To discover more about SLC31A1’s biofunction, we used GO and KEGG pathway enrichment analyses on its 7 targeted binding proteins, which revealed that BP was involved in copper ion transport and cellular copper ion homeostasis. The MF was primarily involved in copper ion binding.The main pathways were enriched in mineral absorption, indicating that SLC31A1 regulates cellular copper levels by controlling cellular uptake or efflux of copper^50,51^.

It has previously been described that ncRNAs, including miRNAs and lncRNAs, play a role in gene regulation by communicating the ceRNA mechanism^52,53^. MiR-195-5p has been discovered to act as tumor-suppressive miRNAs in BC and 11 other types of human tumors^54^, which may be the most promising regulatory miRNA of SLC31A1 in BC. It may mediate its effects by simultaneously inhibiting multiple pro-neoplastic factors in each of these cancers^55^.

The putative lncRNAs of the miR-195-5p/SLC31A1 axis should be carcinogenic in BC^56^. The upstream lncRNAs of the miR-195-5p/SLC31A1 axis were predicted. Two of the most likely upregulated lncRNAs, SNHG16 and AC021092.1, were discovered using expression analysis and correlation analysis. SNHG16 has been implicated as an oncogene in various cancers, including BC. For instance, SNHG16 expression is up-regulated in BC and induces BC cell migration^57^. SNHG16/miR-195-5p/SLC31A1 axis wsa recognized as a candidate regulatory mechanism in BC.

Based on the aforementioned research, we evidenced through a series of crucial in vivo and in vitro investigations that SLC31A1 significantly impacts the promotion of Her2 + enriched BC cells.

There are a few limitations to our study. On the one hand, the Cuproptosis-related gene prognostic signature is based on the online TCGA and GTEx data. There was no relevant external data from other public databases to validate the prediction model, the functions and phenotypes of SLC31A1. On the other hand, our results need further verification in BC through clinical studies and functional analysis, and mechanistic studies are significant.

## Conclusion

In summary, in the present study, our results systematically demonstrated the expression and prognostic value of Cuproptosis-related genes in BC and its key gene SLC31A1 to guide diagnosis and treatment for BC. In addition, we constructed a prognostic risk signature, which could be used as an independent prognostic biomarker to predict the prognosis of patients with BC. SLC31A1 potentially be used for a precision oncology biomarker or therapeutic target.

## Supplementary Information

Supplementary Material 1.

Supplementary Material 2.

Supplementary Material 3.

Supplementary Material 4.

Supplementary Material 5.

## Data Availability

The datasets generated and analysed during the current study are available in the TCGA repository (https://portal.gdc.cancer.gov/), GEO (GSE65212, http://www.ncbi.nlm.nih.gov/geo).

## Abbreviations

BC: Breast cancer
KM: Kaplan–Meier
IDC: Infiltrating Ductal Carcinoma
OS: Overall survival
THPA: The Human Protein Atlas
TCGA: The Cancer Genome Atlas
GTEx: Genotype Tissue Expression
GEO: Gene Expression Omnibus
PPI: Protein-protein interaction
GO: Gene Ontology
KEGG: Kyoto Encyclopedia of Genes and Genomes
DE: Differentially expressed
CTL: Cytotoxic T-cell levels
TIDE: Tumor Immune Dysfunction and Exclusion
ICB: Immune checkpoint blockade

## Declarations

All animal experiments complied with ARRIVE guidelines and were performed in accordance with the National Institutes of Health guide for the care and use of laboratory animals (NIH Publication No. 8023, revised 1978).
