# Supplementary figures and images for: Identification of SLC31A1 as a prognostic biomarker and a target for therapeutics in breast cancer
Source: Sci Rep. 2024 Oct 24;14:25120. doi: 10.1038/s41598-024-76162-x (PMC11502855; doi:10.1038/s41598-024-76162-x)

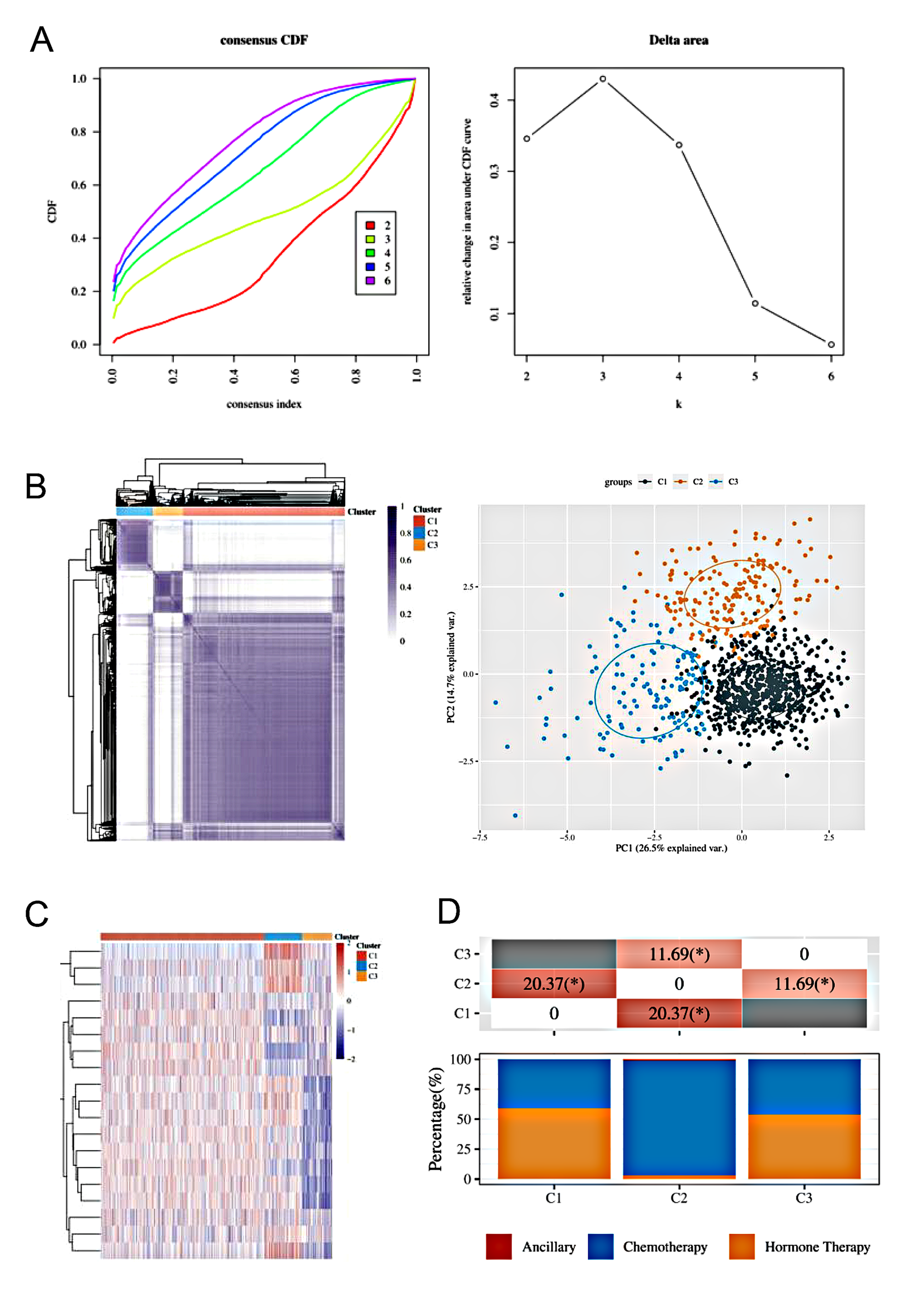

Supplement: Supplementary file 1 — Supplementary Material 1. [file 41598_2024_76162_MOESM1_ESM.tif]

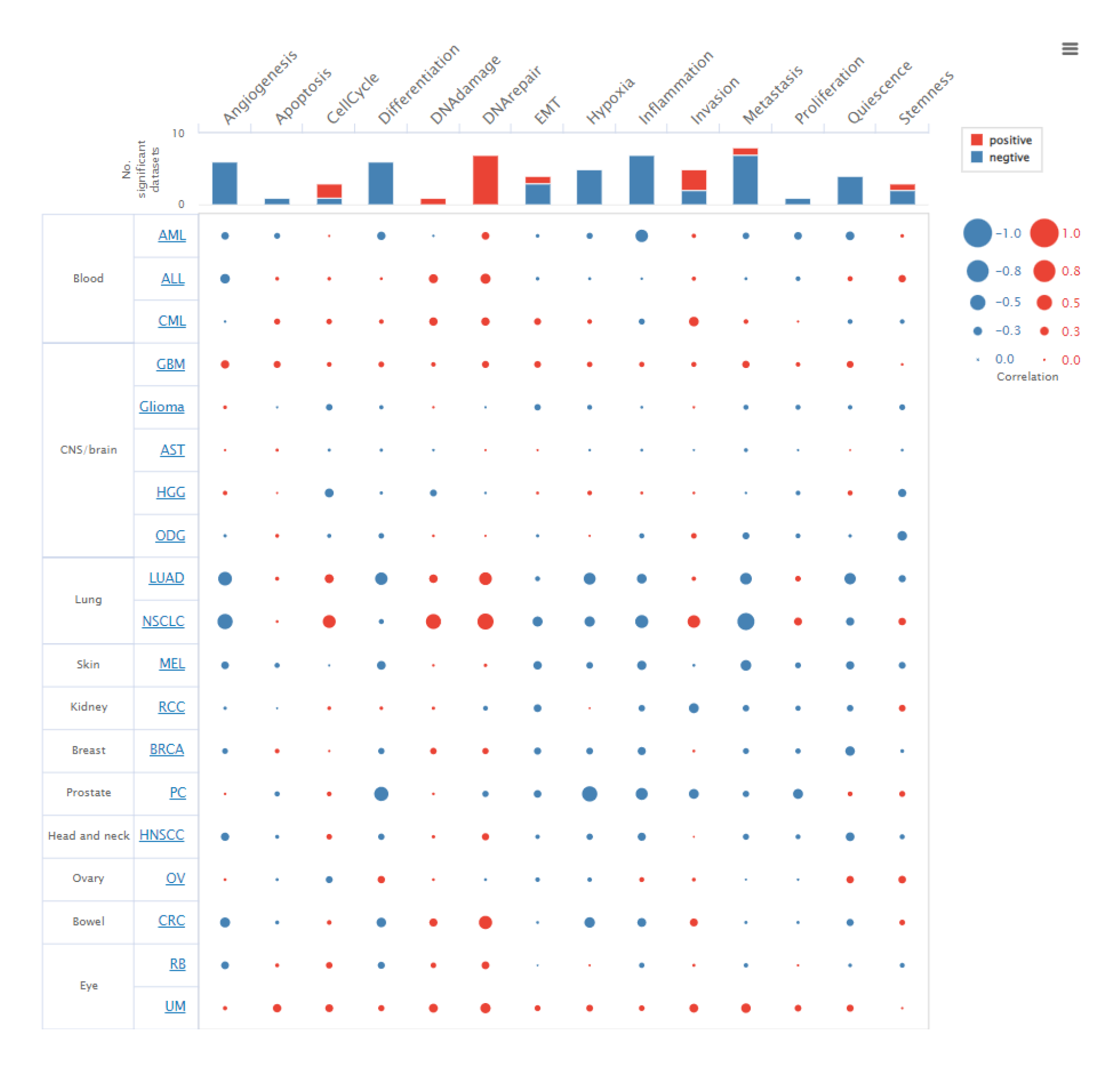

Supplement: Supplementary file 2 — Supplementary Material 2. [file 41598_2024_76162_MOESM2_ESM.tif]

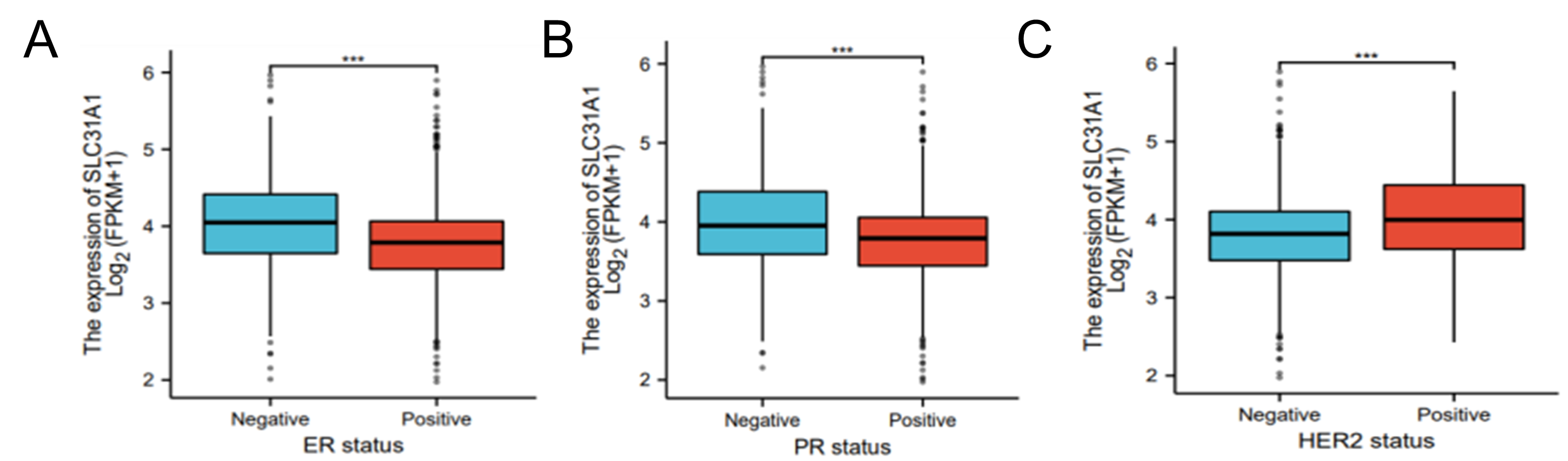

Supplement: Supplementary file 3 — Supplementary Material 3. [file 41598_2024_76162_MOESM3_ESM.tif]

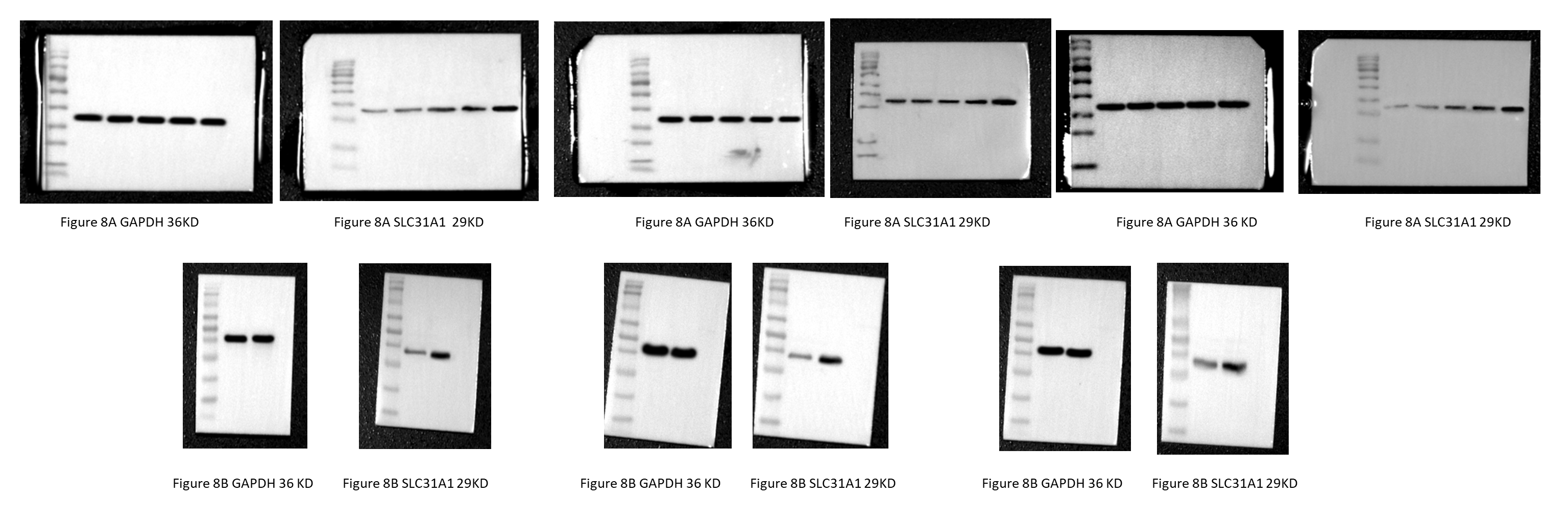

Supplement: Supplementary file 5 — Supplementary Material 5. [file 41598_2024_76162_MOESM5_ESM.tif]
