# Supplementary material for: Identification of SLC31A1 as a prognostic biomarker and a target for therapeutics in breast cancer
Source: Sci Rep. 2024 Oct 24;14:25120. doi: 10.1038/s41598-024-76162-x (PMC11502855; doi:10.1038/s41598-024-76162-x)
Supplement: Supplementary file 4 — Supplementary Material 4. [file 41598_2024_76162_MOESM4_ESM.docx]

Supplementary figure 1. Construction and comparison of two clusters of BC by Cuprotosis-related genes. A. Consensus clustering CDF for k = 2-6. B. Consensus clustering matrix for k = 3. C. The expression heatmap of Cuprotosis-related genes in 3 subgroups, red represents high expression, and blue represents low expression. D. Distribution of clinical characteristics in samples from different groups. ​Marked with * indicates a significant difference in the distribution of the clinical features between the two groups (*p* <0.05). In the C2 group, significantly more patients received chemotherapy than in the other two groups. BC, breast cancer; CDF, cumulative distribution function.

Supplementary figure 2. According to single-cell studies, SLC31A1 is principally involved in controlling proliferation, DNA repair and apoptosis, especially in BC. BC, breast cancer.

Supplementary figure 3. The expression of SLC31 is lower in ER+ (A) and PR+ (B) BC, and higher in HER2+ (C). BC, breast cancer.*p < 0.05, **p < 0.01,***p < 0.001.
